# Consciousness, Free Energy and Cognitive Algorithms

**DOI:** 10.3389/fpsyg.2020.01675

**Published:** 2020-07-30

**Authors:** Thomas Rabeyron, Alain Finkel

**Affiliations:** ^1^Psychology Department, Université de Lorraine, Nancy, France; ^2^Psychology Department, University of Edinburgh, Edinburgh, United Kingdom; ^3^École normale supérieure Paris-Saclay, Cachan, France

**Keywords:** consciousness, entropy, free energy, algorithms, subjectivicty

## Consciousness Studies: From the Bayesian Brain to the Field of Consciousness

Different theoretical approaches have tried to model consciousness and subjective experience, from phenomenology (Husserl, 1913), cognitive psychology and neuroscience (Baars, 2005; Dehaene, 2014), artificial intelligence and cybernetics (Baars and Franklin, 2009; Rudrauf and Debban, 2018), statistical physics and probabilistic models (Solms and Friston, 2018) to mathematics of relationships (Ehresmann and Vanbremeersch, 2009). In this regard, even if the neurobiological functioning of the brain is different from the symbolic processing of a computer (Varela et al., 1991), it might be relevant to conceptualize psychological activity as a Turing machine. For example, Dehaene (2014) assumes that this type of machine offers “a fairly reasonable model of the operations that our brain is capable of performing under the control of consciousness” (p. 151) and points out that “the conscious brain (...) functions like a human Turing machine that allows us to mentally execute any algorithm^1^. Its calculations are very slow, because each intermediate result must be stored in working memory before being sent to the next step—but its computing power is impressive” (p. 150). From this point of view, we would like to suggest in this paper how we could rely on fundamental tools used in computer sciences such as computability theory, algorithmic and finite automata (Pin, 2006; Wolper, 2006) in order to improve our understanding of consciousness.

Among current theories of consciousness, one of the most promising has been developed during the last 10 years by Karl Friston (2009) which states that the brain constructs a predictive representation of its environment that infers the probable causes of sensory stimuli. This representation, or simulation, would lead, or could even be equal, to consciousness (Solms, 2013). This predictive model cannot be right all the time and sometimes there must be a “gap” between the probabilistic representation of the world produced by the brain and the actual perceptual data coming from the environment. It engenders an increase of entropy and free energy in the brain, which would induce subjective feelings of surprise (Friston, 2009; Carhart-Harris and Friston, 2010). Thus, to reduce entropy and free energy, the brain improves progressively its Bayesian probabilistic model of the potential cause of its sensations based on previous assumptions.

Continuing the Bayesian brain hypothesis and Friston's work, Rudrauf and his colleagues (Rudrauf and Debban, 2018; Williford et al., 2018) recently introduced the “Projective Consciousness Model” (PCM) which is a projective geometrical model of the perspectival phenomenological structure of the field of consciousness^2^. The PCM accounts for “the states of the agent's body in its relations to the world and to others by being constantly quantified by the processes of active inference” (Rudrauf and Debban, 2018, p. 161). Its main function is to reduce free energy and “realize a projective geometrical rendering engine embedded in a general active inference engine, which in turn is presided over by a global free energy minimization algorithm” (Williford et al., 2018, p. 9). The PCM is more precisely composed of a (1) “World Model,” mainly unconscious, which stores in memory all the agent's prior beliefs and generative models (2) the “Field of Consciousness” (FoC) which is an explicit model of subjective and conscious experience which takes the form of a simulation in three-dimensions. The FoC represents the sensory perceptions and scenes imagined at any given moment with a specific point of view and can be studied thanks to a domain of mathematics called projective geometry.

## Modeling of the Subjective Experience Using Cognitive Analysis

Complementary to these computational approaches and “third person point of view” of brain functioning, methods inspired by phenomenology have been developed—explicitation interview (Maurel, 2009; Vermersch, 2012) or micro-phenomenology (Petitmengin and Bitbol, 2009; Bitbol and Petitmengin, 2017)—in order to improve our understanding of subjective experience from the “first-person point of view^3^.” One of these neurophenomenological approaches called Cognitive Analysis (CA) has been recently developed by Finkel (1992, 2017) and Tellier and Finkel (1995). CA uses specific interview techniques and modeling tools aimed at describing subjective experience (Finkel, 2017). It also differs from other neurophenomenological approaches by relying on research conducted on mental representations (Kosslyn and Koenig, 1995; Pearson and Kosslyn, 2013) and by using tools from fundamental computer sciences (finite automata and algorithms) following in particular the work of Fodor (1975, 1979, 1983).

CA permits a precise description of the succession of representations used by an individual in order to get closer to his subjective experience (Finkel, 2017). Mental activity is broken done more precisely into three main types of mental objects: sensations (visual, auditive, or kinesthetic), emotions (primary and secondary) and symbolic (verbal language). These mental objects “appear” within the attentional buffer which is itself connected to a long-term information storage system. The subjective experience will also rely on the attentional processes that can be focalized on the internal or the external world. The stream of consciousness can then be conceptualized as a cognitive algorithmic sequence, i.e., a finite sequence of internal and external states and actions (Finkel and Tellier, 1996). Subjective experiences of variable complexity can be analyzed in this way, whether they concern a simple phenomenological experience (e.g., recalling a lived scene), a simple cognitive task (e.g., an addition) or a more complex phenomenological experience (e.g., an Out of Body Experience, see Rabeyron and Caussie, 2016). We thus obtain an algorithm which is a synthetic representation of the successive mental states and the actions carried out during each of these states.

The detailed analysis of a sequence lasting a few seconds sometimes require an interview lasting several hours (Rabeyron, 2020), underlying the incredible density of mental representations and operations that characterize conscious and subjective experience. These cognitive algorithms represent an extremely fast succession of representations concerning the internal and the external world composed of sensations, emotions and words. From this point of view, it is interesting to note that the degree of self-reflexivity of the subject is often limited toward his own mental processes. This is the consequence of the speed with which the representations follow one another and the fact that the subject usually pays limited attention to them during ordinary states of consciousness^4^. It is also possible to compare several interviews with the same individual in order to identify recurring patterns and obtain specific cognitive styles (Tellier and Finkel, 1995). This highlights that the same individual usually uses a finite number of algorithms to handle a wide variety of tasks and situations.

## Consciousness, Cognitive Algorithms, and the Reduction of Free Energy

**Figure 1:**
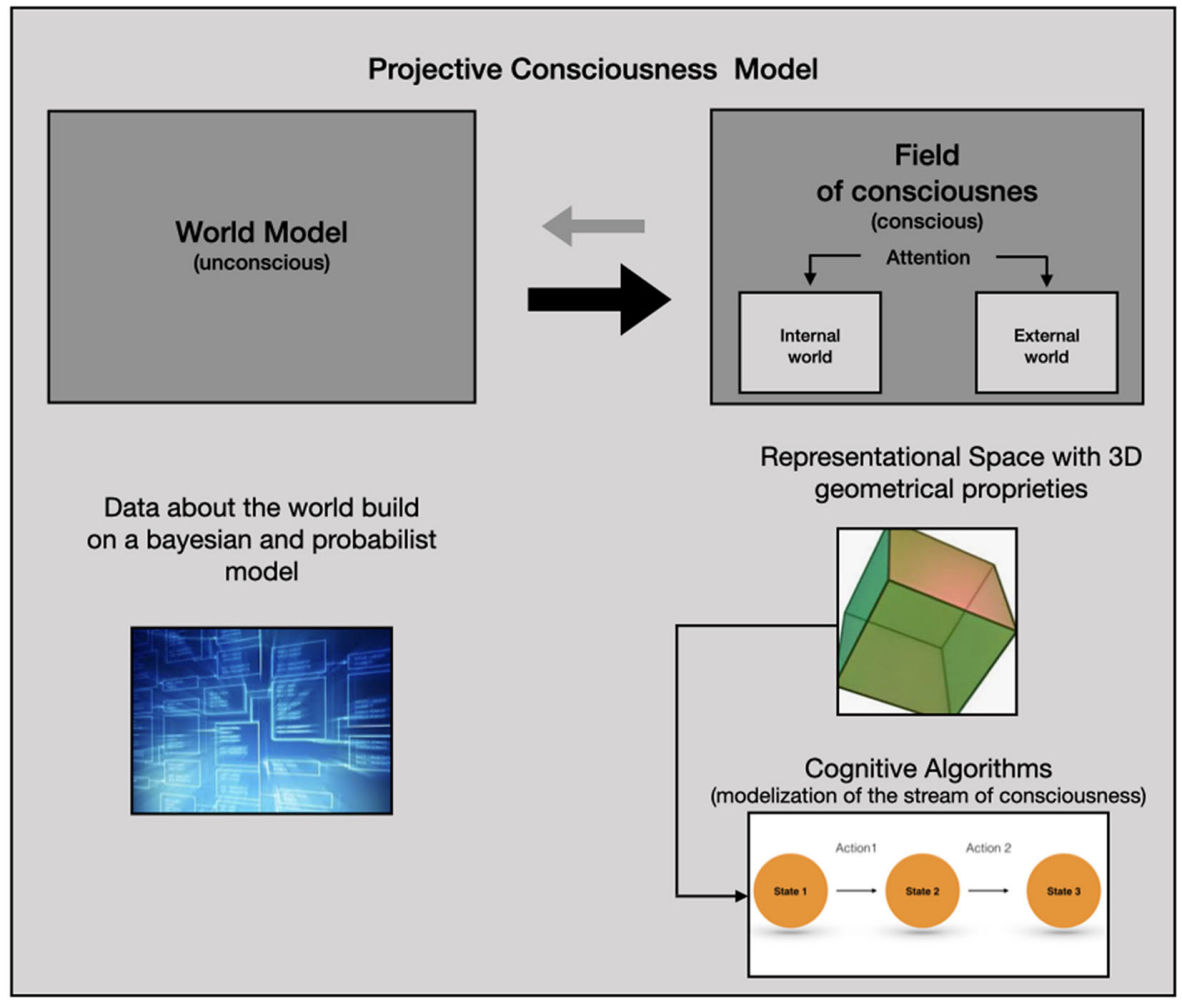
The Projective Consciousness Model (PCM) is made of two components: (1) the world model is a set of data “stored” in the brain concerning the world which is constantly updated according to the subject's interactions with the environment (gray arrow from right to left). (2) the PCM compute a field of consciousness from the World Model on which the subjective experience is produced as a three-dimensional geometric space (black arrow from left to right, which is bolder since the FoC is computed using the World Model data). The content of this field depends on the attentional processes that can be oriented on the internal or the external world. Cognitive analysis describes the cognitive algorithms that characterize the subjective experience in the FoC.

We are now going to describe how PCM and CA could be associated in order to improve our understanding of consciousness. In this regard, we need first to recall that for Williford et al. (2018) “the PCM combines a model of cognitive and affective dynamics based on variational and expected Free Energy (FE) minimization with a model of perspective taking [or a “Field of Consciousness” (FoC) embedding a point of view] based on 3D projective geometry”(p. 2). From this point of view, we can consider that the brain produces a “virtual reality” whose fundamental function is to help the individual to interact with its environment in order to reduce entropy and free energy (Hobson et al., 2014). What is described by Williford et al. (2018) can be conceived as the biological “hardware” necessary to create this virtual reality model—as well as the geometric proprieties of this three-dimensional space—but not the “software” that is used by consciousness to reduce entropy. CA may describe conscious experience in a sufficient detailed manner to determine these “mental softwares” or “mental programs.” We thus hypothesize that the brain integrates and develops specifics cognitive algorithms in order to reduce free energy. A synthesis of these different elements is proposed in Figure 1.

These algorithms can concern all the behaviors and mental functioning. For example, experts in any field (a scientist, a football player, a pilot, etc.) will be rarely surprised by new events because they have the ability to anticipate their environment thanks to these complex and reliable algorithms. Consequently, the gap between their internal representations and the actual states of the world is very limited and the resulting free energy induced by the environment decreases (i.e., a pilot is able during an accident to use specific cognitive algorithms composed of mental representations and physical behavior that he will apply in an efficient manner thanks to his training). Given that “a key function of a mind/brain is to process information so as to assist the organism that surrounds it in surviving, and that a successful mind/brain will do so as efficiently as possible” (Wiggins, 2018, p. 13), individuals will thus naturally tend to improve the quality and the complexity of their cognitive algorithms in order to increase their adaptive abilities. We propose that a research program based on the analysis and modeling of these algorithms could lead to promising empirical discoveries in these four directions:

1/The notion of “borrowed brain” has been proposed to describe how the infant internalizes the Bayesian processes of attachment figures (Holmes and Nolte, 2019). Similarly, cognitive algorithms are probably internalized during infancy from the attachment figure's own cognitive algorithms. It could be relevant to study the different cognitive algorithms used, and probably shared, by the same members of a family and especially between the children (at different ages) and their parents.

2/Propose a “genealogy” of the development of these algorithms, which take rudimentary forms during infancy—initially focused on emotional and body experiences—to very complex versions in adulthood relying notably on words. These algorithms are probably developed according to a process of increasing complexity and metaphorization as it has been shown for language (Lakoff and Johnson, 2003; Lakoff, 2014).

3/Develop a psychological test to determine precisely which algorithms are usually used by an individual. This approach can be developed, for example, to study common patterns appearing in decision-making (Tellier and Finkel, 1995). We could also “extract,” in a novel manner, the cognitive algorithms used by experts in a given domain in order to better transmit them during training programs as it has already been carried out with explicitation interviews (Maurel, 2009). In this regard, clinical applications have also been developed recently in neuropsychology, clinical psychology and psychiatry relying on neurophenomenological explorations of subjective experiences (Petitmengin, 2006).

4/Evaluate the relevance of these cognitive algorithms in terms of free energy regulation as an extension of the work developed by Rudrauf and Debban (2018). These algorithms might be a “missing link” concerning the understanding of how PCM reduces free energy. We also join the hypothesis developed in the IDyoT model (Wiggins and Forth, 2015) which relies on the “the key idea that the biggest reduction in entropy corresponds with the maximum information gain, and so the most efficient decision tree is the one that repeatedly makes the biggest possible information gain first” (Wiggins, 2018, p. 14). From this point of view, creativity could be conceived has the ability to produce original cognitive algorithms whose main function would be information efficiency and thus the reduction of free energy. Compared to the IDyoT model, CA concerns a meta-level of information treatment because it analyzes the components of the subjective experience and not the way these components have been initially produced by the brain.

These four research perspectives, relying on recent development of the Bayesian brain models and Cognitive Analysis, might open innovative perspectives both in terms of research and clinical applications. It could also help to diminish the current gap (Lutz and Thompson, 2003) in our knowledge between the first-person and the third-person point of views concerning our understanding of consciousness and subjectivity.

## Author Contributions

TR wrote the first draft of this paper and AF improved this draft. AF has created the CA and has described its principles. TR proposed the idea that cognitive algorithms could reduce free energy. All authors contributed to the article and approved the submitted version.

## Conflict of Interest

The authors declare that the research was conducted in the absence of any commercial or financial relationships that could be construed as a potential conflict of interest.

## Footnotes

^1^We do not assume that the brain is a computer—as proposed in the symbolic approach whose weaknesses opened the way to connectionism (Varela et al., 1991)—but we suppose that we can analyze part of its functioning using original tools borrowed from the field of computer studies.

^2^From a more philosophical point of view concerning how the PCM could be articulated with the hard problem of consciousness (Chalmers, 1996) and the infinite regress argument (Dennett, 1991), see Williford et al. (2018), especially on p. 10–14.

^3^About the reliability and the validity of such a neurophenomenological approach, see in particular the arguments proposed by Petitmengin (2009).

^4^The degree of reflexivity can be limited during a task but the neurophenomenological approach makes it possible to access afterwards what happened during the task. The participants are usually very surprised to discover what happened in their mind without noticing it during the task itself, because the stream of consciousness is particularly rich and fast. In other words, they were conscious but they were not “thinking about their thinking” during the task itself.
